# Clinical and Basic Research on Renshen Yangrong Decoction

**DOI:** 10.3389/fnut.2019.00175

**Published:** 2019-12-02

**Authors:** Wei Sheng, Yun Wang, Jiang-Bo Li, Hua-Shan Xu

**Affiliations:** ^1^School of Mental Health, Bengbu Medical College, Anhui, China; ^2^Department of Psychiatry, Fourth People's Hospital of Xuancheng, Anhui, China; ^3^Department of Psychiatry, People's Hospital of Wuhan University, Wuhan, China; ^4^Department of Clinical Psychology, Wannan Medical College, Second People's Hospital of Wuhu, Anhui, China

**Keywords:** Renshen Yangrong Decoction, hematopoiesis, immune function, pharmacology, clinical treatment

## Abstract

Renshen Yangrong Decoction has been used to treat asthenic disease symptoms, such as exhaustion, and qi and blood deficiency diseases. It not only promotes hematopoietic function and improve immune functions, but also alleviates coronary heart diseases, diabetic complications, malignant tumor, and brain injury. It has satisfactory curative effect on sleep disorders and fatigue. Herein, we provide an overview of fundamental research on Renshen Yangrong Decoction focusing on its hematopoietic and immune functions and the status of clinical research with regard to the above-mentioned diseases in recent years.

## Introduction

### History of Renshen Yangrong Decoction (RYD)

RYD, previously known as yangrong decoction, was originally prepared by Dr. Chen Yan, a doctor belonging to the Southern Song Dynasty. This traditional Chinese herbal prescription has been recorded in the book “Treatise on Three Categories of Pathogenic Factors,” written in 1174. Subsequently, this decoction was named “Renshen Yangrong Decoction” in the book “Prescriptions of the Bureau of Taiping People's Welfare Pharmacy.”

### Composition and General Pharmacological Action of RYD

The decoction is composed of ginseng, astragalus, *Atractylodes macrocephala, Poria cocos, Angelica* sp., radix paeoniae alba, processed rehmannia root, dried tangerine or orange peel, cortex cinnamomi, *Schisandra chinensis, Polygala* sp., and licorice. In traditional Chinese medicine, this decoction is prescribed to treat conditions such as fatigue; qi and blood deficiency; lusterless complexion; laziness; tastelessness; limb stagnation; bone and flesh ache; cough and asthma heart blood weakness; heart palpitation; pharynx and lip dryness; spontaneous perspiration; night sweating; chest, palm, and sole dysphoria [fever]; chilly sensation; cold limbs; and weak pulse (1). However, the diseases in Western medicine for which RYD could be suitable have not been clearly identified. Pharmacological studies on the ingredients of RYD have shown that ginseng can promote the proliferation of hematopoietic cells in the bone marrow and increase white blood cells, red blood cells, and hemoglobin in the peripheral blood (2). *Astragalus membranaceus* has dual immunomodulatory activity (3). *Atractylodes macrocephala* can promote cellular immune function (4). *Poria coco* can enhance immunity and exert anticancer and cytotoxic effects (5). *Angelica sinensis* has anticoagulant and inhibitory effects on fibrous tissue proliferation (6). *Rehmannia glutinosa* was reported to promote the proliferation and differentiation of mouse pluripotent hematopoietic stem cells and significantly increase the number of erythroid colonies (7). *Schisandra chinensis* can increase white blood cell count, enhance immunity, and exert anticancer effect (8). *Polygala* sp. enhances immunity (9). Tangerine peel can antagonize the mutagenicity of a variety of chemotherapeutic drugs, such as fluorouracil, thiothiazine, and cyclophosphamide, and can significantly protect germ cells from damage induced by cyclophosphamide in male mice (10). Licorice exerts antiviral, lipid-lowering, anti-atherosclerotic, detoxifying, anti-arrhythmic, anti-tumor, and anti-oxidation effects (11). Herein, we summarize the basic properties of RYD and its clinical application in promoting hematopoiesis and immune function.

## Bioactivities of RYD

### Promotion of Hematopoiesis

A previous study, which evaluated the effect of RYD on human peripheral blood mononuclear cells, reported that RYD can promote the proliferation of granulocyte-macrophage colony-stimulating factor. Compared with monotherapy, butylopinin combined with RYD significantly promoted the colony formation ability of granulosa cells and improved hematopoietic function after chemotherapy (12). Bao et al. reported that RYD does not directly act on T lymphocyte subset (CD3 and CD4)-positive cells but improves hematopoiesis in the presence of antigen-presenting cells. Moreover, it stimulates the activity of monocytes, promotes an increase in splenocytes, and stimulates the recovery of erythrocytes. It can also stimulate the production of colony stimulating factor, promoting the formation of IL-6 and differentiation of lymphocytes, monocytes, and granulocytes (13). A study on the effects of RYD in a bone marrow suppression mice model indicated that it can promote the recovery of hematopoietic system function, thus improving hematopoiesis and increasing the number of peripheral blood cells and marrow nucleated cells and the hematopoietic area of the marrow. It also stimulated the colony formation ability of hematopoietic stem/progenitor cells (erythrocytes, granulocytes, and megakaryocytes) cultured *in vitro*. This resulted in reduction of early and late apoptosis in hematopoietic stem/progenitor cells, accelerating the proliferation, and differentiation of hematopoietic stem/progenitor cells, unblocking the DNA pre-synthesis phase (G1 phase), and shortening the transformation cycle of hematopoietic stem/progenitor cells to active phase (phase S and G2/M). RYD is mainly involved in the regulation and functional recovery of the hematopoietic system in mice with bone marrow suppression through the above pathway, thus promoting hematopoietic function (14).

### Regulation of Immune Function in Mice

It has been proved that RYD can enhance the cytotoxic activity of T lymphocytes, thereby increasing the number of CD4+ and CD8+ T cells and exerting a positive regulatory effect on the production of immunoglobulin G in immunodeficient mice; cyclophosphamide was used to inhibit the immune function of mice as a reference. It has also been confirmed that RYD as a traditional Chinese medicine prescription can activate and enhance the function of non-specific natural killer cells to improve cellular and humoral immunity (15). It also significantly enhances the lymphocyte transformation rate in mice (16). Ten patients with herpes zoster were randomly divided into two groups: the control group received basic antiviral treatment and the treatment group received RYD and basic antiviral treatment simultaneously. A comparison of time required for pain relief and recurrence after 3 months between the two groups revealed that the time required for relieving herpes zoster neuralgia in the treatment group was significantly shorter than that in the control group, and there was no recurrence of herpes zoster neuralgia after 3 months in the treatment group. Thus, it is believed that RYD can improve immune function and effectively relieve and prevent herpes zoster neuralgia (17).

## Application of RYD in the Treatment of Various Diseases

### Application of RYD in Treatment of Coronary Heart Diseases and Diabetic Complications

The various stages of coronary heart diseases have a close relationship with qi and blood. According to Prof. Lin Huijuan's theory of “Qi and blood coordination leads the heart vessel run,” Zhan et al. used RYD to regulate qi and blood to treat coronary heart diseases. Good clinical efficacy was observed in the treatment of subclinical angina, myocardial infarction, and ischemic cardiomyopathy of coronary heart diseases (18, 19). Cong reported that RYD had lesser adverse effects upon long-term administration to 17 patients with ischemic heart disease (administration for more than 8 weeks). The results showed that RYD can inhibit platelet activity, suggesting its antiplatelet aggregation effect; therefore, RYD can be used to treat and improve the clinical symptoms of ischemic heart diseases (20). The “HeXue ShengLuo Recipe” Chinese herbal prescription combination of astragalus, ginseng, prepared rehmannia root, angelica, radix paeoniae, ligusticum (chuanxiong), and *Rhodiola* sp. can promote the proliferation, migration, and DNA synthesis of human umbilical vein endothelial cells, suggesting that it promotes angiogenesis. It might be one of the mechanisms by which RYD prevents and treats ischemic heart disease (21). Sixty patients with diabetic foot ulcers were administered blood sugar control and anti-infective therapy according to their condition, patients all with systemic infection control. The patients with effective external treatment for foot wound were randomly divided into the control and treatment groups. The treatment group was additionally administered oral RYD, and the clinical indicators were observed and compared with those of the control group, such as meat purification time, granulation flat surface time, epithelialization time, wound healing time, and serum albumin level. The results showed that RYD significantly increased the serum albumin level, improved the nutritional status of patients, and shortened the healing time (22). Another study has shown that RYD has a positive effect on self-sensory somatic symptoms of diabetes such as cold sensation, numbness, vertigo, general fatigue, and limb pain. Moreover, a significant difference was observed before and after treatment, indicating that RYD had a positive effect on the improvement in somatic symptoms in patients with diabetes (23).

### Application of RYD on the Clinical Treatment of Malignant Tumor

Currently, the toxicity and adverse reactions of radiotherapy and chemotherapy in the clinical treatment of malignant tumor are important factors that lead to anorexia, emaciation, weakness, and even shorten the survival period. Bone marrow suppression mainly causes leukocytopenia, thrombocytopenia, and erythrocytopenia, leading to weakness, exhaustion, and immunity loss, affecting the quality of life and prognosis of cancer. A pharmacological study of RYD in the treatment of lung cancer patients with qi-yin deficiency revealed that the decoction constituents radix astragali, ginseng, A*ngelica sinensis*, and radix rehmannia preparata can activate the immune system, increasing the ratio of T lymphocyte and improving the immune function of tumor cells. Astragalus, *Codonopsis pilosula*, and *Atractylodes macrocephala* can enhance phagocytosis ability of the reticuloendothelial system and exert inhibitory effect on T suppressor cells, thereby enhancing hematopoiesis. *Poria cocos* and *Angelica sinensis* have an obvious anticancer activity and can promote immunity. *Schisandra chinensis* can inhibit the proliferation of cancer cells and improve immunity (24). Zeng Jiao Fei administered chemotherapy drugs and RYD to nude mice with gastric cancer, and then analyzed two T cell subsets and quantified the TNF-α level and spleen and thymus indices. The results revealed that RYD can enhance the immune function of mice after chemotherapy by regulating the expression of T cell subsets and TNF-α in the blood and ameliorate thymus and spleen atrophy in mice that received chemotherapy of varying degrees, thereby improving the immune function (25). It can also improve low immunity and emaciation due to tumor growth or adverse effects of radiotherapy and chemotherapy, such as leukocyte reduction, thrombocytopenia, hemoglobin reduction, nausea, vomiting, arrhythmia, hair loss, and fever (26). It can also effectively improve the completion rate of chemotherapy and reduce the incidence of tissue swelling, fatigue, palpitations, and insomnia (27). A recent analysis of curative effect of RYD combined with chemotherapy for advanced lung cancer with deficiency of both qi and yin showed that the total effective rate was 86.67%, with a complete remission rate of 33.33%, partial remission rate of 43.33%, mild remission rate of 10%, and stability rate of 13.33%. Furthermore, the curative effect of RYD combined with chemotherapy was better than that of chemotherapy alone (24). A study of the effects of RYD combined with chemotherapy for advanced lung cancer showed that short-term (2 months) treatment led to complete remission in 8 cases (25%), partial remission in 10 cases (31.25%), no change in 9 cases (28.13%), and deterioration in 5 cases (15.62%), and the effective rate was 56.25%, which was better than that of the control group. The long-term survival rate of the treatment group was 100% (32 cases) in 1 year, 78.13% (25 cases) in 2 years, and 43.75% (14 cases) in 3 years, and the annual survival rate was better than that in the control group (28). These data suggest that short- or long-term combination treatment with chemotherapy and RYD can better relieve the clinical symptoms and improve the survival rate. Research on the application of RYD in the treatment of breast cancer revealed that long-term use of RYD after chemotherapy can improve immunity and hematopoietic function to some extent. Improvement in the quality of life, suppression of bone marrow, and alleviation in nausea, vomiting, and other gastrointestinal reactions in the treatment group were better than those in the control group. The results suggest that RYD can reduce toxic reactions induced by chemotherapy and improve the quality of life in patients with breast cancer (29). Some studies have investigated the effect of ginseng yangrong decoction on immune function in lung cancer patients with qi-yin deficiency undergoing chemotherapy. It has been reported that ginseng yangrong decoction can effectively improve the short-term clinical efficacy, improve the quality of life and immune function, and reduce the occurrence of toxic and adverse effects in patients (30).

### Therapeutic Effect of RYD on Trauma

Renshen Yangrong Decoction can effectively improve the degeneration and loss of cortical and hippocampal neurons in rats with craniocerebral trauma. Through TGF-β1/Smad signaling pathway, RYD can increase the serum levels of decorin, TGF-β1, Smad2, and Smad3. Inflammatory mediators stimulate tissue factors to inhibit the inflammatory reaction caused by craniocerebral injury and improve the degree of traumatic brain injury (31). Previously, rats with open abdomen and abdominal suture were divided into normal and low nutrition groups and administered RYD before operation, after operation, and before and after operation. Daily biochemical determination of serum amino acids and measurement of body weight showed that RYD can improve wound healing disorder caused by low protein and reduce postoperative emaciation with preoperative administration. Furthermore, RYD was found to be effective in rats subjected to low nutrition treatment, preventing wound healing complications and recovering physical strength after operation (32).

### Therapeutic Effects of RYD in Insomnia

Insomnia is a common sleep disorder, which is mainly caused by social, psychological, and awakening disorders. It is believed that deficiency of both qi and blood can cause various clinical symptoms such as less-lazy statement, fatigued spirit, weakness, palpitation, spontaneous sweating, vertigo, and pale or sallow complexion. Yingying carried out a control study in 68 patients with insomnia due to qi and blood deficiency and reported that the total effective rate of the observation group was 94.12%, which was significantly higher than that of the control group (82.36%), and the difference was statistically significant (*P* < 0.05). Thus, RYD can significantly improve the symptoms of insomnia and quality of life of patients with the deficiency of qi and blood. Furthermore, studies have shown that the prescription is safe and painless, with limited adverse effects (33). One hundred and four patients with COPD plateau and sleep disorders were randomly divided into treatment (54 patients) and control groups (50 patients). The control group was administered alprazolam tablets and the treatment group was administered the coordinate ginseng glory tonga subtraction treatment. After 2 weeks, the two groups were observed for clinical curative effect and changes in Athens insomnia scale (AIS) score. The results revealed that the total effective rate of the treatment group was 88.89%, which was significantly higher than that of the control group (74.00%, *P* < 0.01). It can be concluded that ginseng yangrong decoction with or without treatment COPD stable period has a significant effect in patients with sleep disorder (34).

### Therapeutic Effect of RYD on Fatigue

Renshen Yangrong Decoction is a representative prescription for the treatment of asthenic overstrain. Studies have shown that RYD plus Xiaoyao San can significantly improve fatigue symptoms in patients with chronic fatigue syndrome (35). Geng et al. chose 31 patients with chronic fatigue syndrome to observe the therapeutic effects of RYD. The results showed a significant curative effect in 20 patients (64.5%), effective in 8 patients (25.8%), and invalid in 3 patients (9.7%), and the total effective rate was 90.3%. This suggests that RYD has a significant effect in patients with chronic fatigue syndrome (36). Experiments in mice have showed that RYD can improve fatigue resistance, hypoxia tolerance, and high and low temperature resistance, and alleviate fatigue and stress (37). Moreover, RYD can alleviate fatigue symptoms in patients with advanced lung cancer receiving chemotherapy, thus improving the overall quality of life, physical function, and dyspnea (38).

### Therapeutic Effect of RYD on Dementia

Yamamoto divided 37 patients with Alzheimer's dementia into two groups: the experimental group comprised 27 patients who were administered RYD and the control group comprised 10 patients who received bifemelane. The results showed that the overall improvement rate after 8 weeks was 55.6% in the experimental group and 33.3% in the control group. In the experimental group, emotional disturbance, anxiety, irritability, and other somatic symptoms were improved significantly. Laboratory examination showed significant improvements in estrone level, cholinesterase activity, serum total protein level, red blood cell count, and body weight. Therefore, RYD is effective in treating patients with Alzheimer's dementia with low estrone level (39).

### Other Therapeutic Effects of RYD

Adhering to the principle of “homotherapy for heteropathy” and the pathogenesis of qi and blood deficiency, RYD addition and subtraction presented good clinical effects in the treatment of viral hepatitis, insomnia, alopecia, and prolonged menstruation (40). It has satisfactory clinical efficacy in internal medicine (for cold, body weakness, and myocardial ischemia), pediatrics (for infantile chancre syndrome), and gynecologics (for lack of lactation and menopause syndrome) (1). It also has a significant effect in lowering the level of triglyceride and preventing the appearance of low- and high-density lipoproteins (41). Tremor syndrome due to qi and blood deficiency (such as Parkinson's syndrome and Parkinson's disease) is very common in clinical practice. Renshen Yangrong Decoction is a warm supplement prescription suitable for this syndrome. A clinical analysis of the effect of ginseng tonic soup combined with madopar compared with madopar alone for the treatment of syndrome of tremor of qi and blood deficiency has been conducted according to the evaluation criteria of the efficacy of anti-Parkinson drugs. The results showed that RYD combined with madopar can improve somatic symptoms compared with that of madopar treatment alone (42).

## Prospects

Renshen Yangrong Decoction has good clinical effects in the treatment of qi deficiency, blood deficiency, and other symptoms. Considering its hematopoiesis-promoting and immunity-enhancing functions, RYD is promising in the field of western medicine for the treatment of debilitating conditions caused by diseases such as hematological diseases and tumors, expanding its clinical applications. According to inter-individual variability in diseases and physique, adjusting the dosage, and drug composition may have a significant effect on clinical effects. The pharmacological action mechanism of drug components and clinical application for other kinds of diseases with difficulty in diagnosis need to be further studied.

## Author Contributions

All authors assisted in designing the study, preparation of the initial draft of the manuscript, and data collection and interpretation. All authors approved the final version of the manuscript, and all authors agree to be accountable for the content of the work.

### Conflict of Interest

The authors declare that the research was conducted in the absence of any commercial or financial relationships that could be construed as a potential conflict of interest.
